# Efficacy of NAs in combination with Peg-IFN for functional cure in patients with CHB: a meta-analysis of RCTs

**DOI:** 10.1080/07853890.2026.2636319

**Published:** 2026-03-04

**Authors:** Yaqin Zhang, Ziyu Zhang, Xinxin Li, Weihua Cao, Shiyu Wang, Wen Deng, Xin Wei, Linmei Yao, Yao Xie, Minghui Li

**Affiliations:** ^a^ Department of Hepatology Division 2, Beijing Ditan Hospital, Capital Medical University, Beijing, China; ^b^ Department of Hepatology Division 2, Peking University Ditan Teaching Hospital, Beijing, China; ^c^ HBV Infection, Clinical Cure and Immunology Joint Laboratory for Clinical Medicine, Capital Medical University, Beijing, China

**Keywords:** Chronic hepatitis B, interferon, nucleoside analogues, therapeutic effect

## Abstract

**Background:**

The goal of treating chronic hepatitis B (CHB) is to achieve functional cure. We aimed to evaluate the efficacy of the combination of nucleoside (acid) analogues (NAs) and pegylated interferon (Peg-IFN) on the functional cure of CHB patients at the EOT and at the EOF.

**Method:**

PubMed, Embase, and Web of Science were searched systematically up to March 15, 2025. Sixteen RCTs on CHB patients receiving combination or monotherapy were included.

**Result:**

Compared with NAs treatment, the NAs combined with Peg-IFN treatment group significantly improved HBsAg clearance rate (RR: 14.05, 95% CI 6.13–32.20) and HBsAg seroconversion rate (RR: 12.82, 95% CI 5.08–32.33) at the EOT. Moreover, compared with NAs treatment, the NAs combined with Peg-IFN treatment group significantly improved HBsAg clearance rate (RR: 7.70, 95% CI 4.24–13.98), HBsAg seroconversion rate (RR: 11.93, 95% CI 5.14–27.67) at the EOF. However, compared with Peg-IFN monotherapy, the combination therapy group did not show any improvement in HBsAg clearance rate, HBsAg seroconversion rate, and the rate of qHBsAg decrease > 1 log10 IU/mL at the EOT and the EOF. Moreover, in terms of safety, the Peg-IFN monotherapy group showed more ALT flares (ALT > 5 × ULN) during the treatment process.

**Conclusion:**

Compared with NAs therapy, Peg-IFN combined with NAs therapy significantly improves functional cure rate. However, combination therapy shows no additional advantage over Peg-IFN monotherapy in achieving functional cure. The Peg-IFN monotherapy group has a higher incidence of ALT flares than the combination therapy group.

## Introduction

Chronic hepatitis B (CHB) is one of the global public health issues. Approximately 257 million people worldwide are infected with hepatitis B virus (HBV) [1]. Chronic HBV infection can lead to the occurrence of HBV related liver diseases through indirect or direct pathways. Approximately 887000 CHB patients develop severe liver disease (including liver failure, cirrhosis, and hepatocellular carcinoma (HCC)) each year [2]. China has achieved significant progress in decreasing the incidence of HBV infection over the past three decades. This progress can be largely attributed to the implementation of childhood vaccination programs and the extensive coverage of interventions aimed at preventing mother-to-child transmission of HBV, both of which exceed 95%. However, China still faces challenges in achieving the goal of reducing the mortality of hepatitis B by 65% by 2030 [3].

The pursuit of functional cure has become a key treatment endpoint for current and upcoming clinical trials of CHB. Functional cure (also known as clinical cure) refers to the sustained presence of HBsAg negativity (with or without anti-HBs), undetectable HBV DNA levels, and normal liver biochemical parameters after discontinuation of treatment. Moreover, CHB patients can achieve histological improvement after long-term antiviral therapy. At present, the first-line anti-HBV drugs in clinical practice are Peg-IFN α and nucleoside (acid) analogues (NAs). The anti-HBV mechanism of NAs is mainly to inhibit the replication of the virus, thereby reducing the viral load. However, NAs do not eliminate covalently closed circular DNA (cccDNA), leading to viral rebound upon treatment cessation [4]. Therefore, long-term medication is needed to achieve sustained inhibition of virus replication. IFN α can inhibit HBV replication and reduce viral protein production in liver cells through various mechanisms. Patients who respond to IFN α and achieve functional cure exhibit more upregulation of interferon stimulated genes (ISG) [5]. In addition, IFN α also has an overall immunomodulatory effect on CHB patients [6]. However, only a small percentage of patients respond to IFN, and severe adverse reactions result in poor tolerance to IFN treatment [7].

Current research has found that the combination therapy of Peg-IFN and NAs is effective in suppressing HBV DNA replication and reducing HBsAg levels. Recently, various antiviral therapy combination strategies based on NAs and Peg-IFN have emerged, including initial combination therapy [8], NAs sequential Peg-IFN therapy [9], and IFN intermittent therapy [10], to improve th rate of HBeAg loss and HBsAg loss. In 2022, China’s prevention and treatment guidelines for CHB have expanded the antiviral indications, and it is suggested to try to add IFN treatment to the dominant population with NA treatment, which is to prepare for achieving higher rate of functional cure. So the main purpose of this meta-analysis is to evaluate which therapeutic schedule is more advantageous for achieving functional cure.

Consequently, this research seeks to assess the differences in efficacy regarding the attainment of a functional cure between the group receiving Peg-IFN in conjunction with NAs and the monotherapy group among patients with CHB at both the end of treatment (EOT) and the end of follow-up (EOF). Additionally, the study will evaluate the safety profiles of the treatment modalities and investigate the potential risk of recurrence following the cessation of therapy.

## Materials and methods

### Literature retrieval strategy

We searched for relevant studies from the establishment of the database to March 15, 2025 in PubMed, Embase and Web of Science, Utilizing diverse combinations of keywords such as ‘interferon’, ‘chronic hepatitis B’, ‘lamivudine’, ‘Adefovir’, ‘Telbivudine’, ‘Tenofovir’, ‘Tenofovir disoproxil fumarate’, ‘Tenofovir alafenamide fumarate’, ‘Tenofovir amibufenamide’, ‘Entecavir’, ‘randomized controlled trials’, ‘hepatitis B surface antigen’, etc. For detailed information on retrieval strategies, please refer to Supplementary Material 1. In addition, we searched for relevant system reviews to find other papers. The two authors, Yaqin Zhang and Ziyu Zhang, independently conducted the search, review, and selection of all articles. Any discrepancies were addressed through discussions with the third author, Xinxin Li.

### Criteria for inclusion and exclusion

The studies incorporated in this meta-analysis adhere to the subsequent inclusion criteria: (1) The study is a randomized controlled trial(RCT); (2) The study included a treatment strategy of combining NAs with Peg-IFN, with single therapy (NAs or Peg-IFN) as the control group; (3) The Peg-IFN treatment duration in the study was at least 24 weeks; (4) Patients in the study were all over 16 years old; (5) The research report should include at least one of the following outcomes of interest: HBsAg deficiency or HBsAg seroconversion. This research eliminated studies that fulfilled the following exclusion criteria: (1) the study only involved monotherapy; (2) Participants had other types of viral hepatitis, autoimmune hepatitis, and other causes of hepatitis; (3) Subjects with severe liver disease, liver transplantation, or chemotherapy, and (4) studies without primary outcome measures or incomplete data.

### Data extraction

Data were extracted utilizing standardized tables that included study characteristics such as the first author, publication year, study country, study design, treatment group, sample size, baseline HBeAg status, antiviral treatment strategy (Peg IFN and/or NAs regimen), treatment duration, and follow-up duration, as well as participant characteristics (baseline HBsAg level and baseline ALT level).(Supplementary Tables 1 and 2). This process was independently conducted by two authors, Ziyu Zhang and Weihua Cao, and was subsequently reviewed by a third party, Yaqin Zhang.

### Risk assessment of bias

We used the Cochrane Randomized Trial Bias Risk Tool (RoB 2) 2nd edition to assess the risk of bias in included RCTs. Methodological quality is characterized as the credibility of bias in the comparison of interventions that will be limited by the design and reporting of RCTs. The differences are resolved through discussion with a third party (Yaqin Zhang).

### Definition of ending

Evaluate efficacy based on the following criteria: HBsAg clearance at the EOT and the EOF: defined as HBsAg turning negative, regardless of whether anti-HBs are produced; HBsAg seroconversion: HBsAg becomes negative, accompanied by the production of HBsAb. Secondary outcome measure: qHBsAg decrease > 1 log10 IU/ml. Safety evaluation: Diseases with severe liver dysfunction and other reasons that must be withdrawn from the study.

### Statistical analysis

This comprehensive analysis was conducted in accordance with the Preferred Reporting Items for Systematic Reviews and Meta-Analyses (PRISMA) guidelines. This systematic review was prospectively registered with PROSPERO (Registration number: CRD42024571401). The study protocol is publicly available at: https://www.crd.york.ac.uk/PROSPERO/view/CRD42024571401.

Review Manager 5.3 is employed for the purpose of data analysis. All P-values are calculated using a two-tailed approach, with a significance threshold set at 0.05. For prospective studies and binary data, relative risk (RR) was used as the outcome measure and its 95% CI was reported. This meta-analysis employed either a random effects model or a fixed effects model, contingent upon the degree of heterogeneity observed. Evaluate heterogeneity through I^2^ and *P*-values. When the value of I^2^ is less than 50% or 50% less than the value of I^2^ is less than 60%, but *p* > 0.1, the fixed effects method is used. Conversely, the random effects model is utilized when the I^2^ statistic exceeds 60%, or when the I^2^ statistic falls within the range of 50% to 60% with a *P*-value less than 0.1. Additionally, a sensitivity analysis was performed to assess the robustness of the findings. Review Manager 5.3 funnel plot was used to evaluate publication bias.

## Result

### Research and participant characteristics

**Figure 1.**
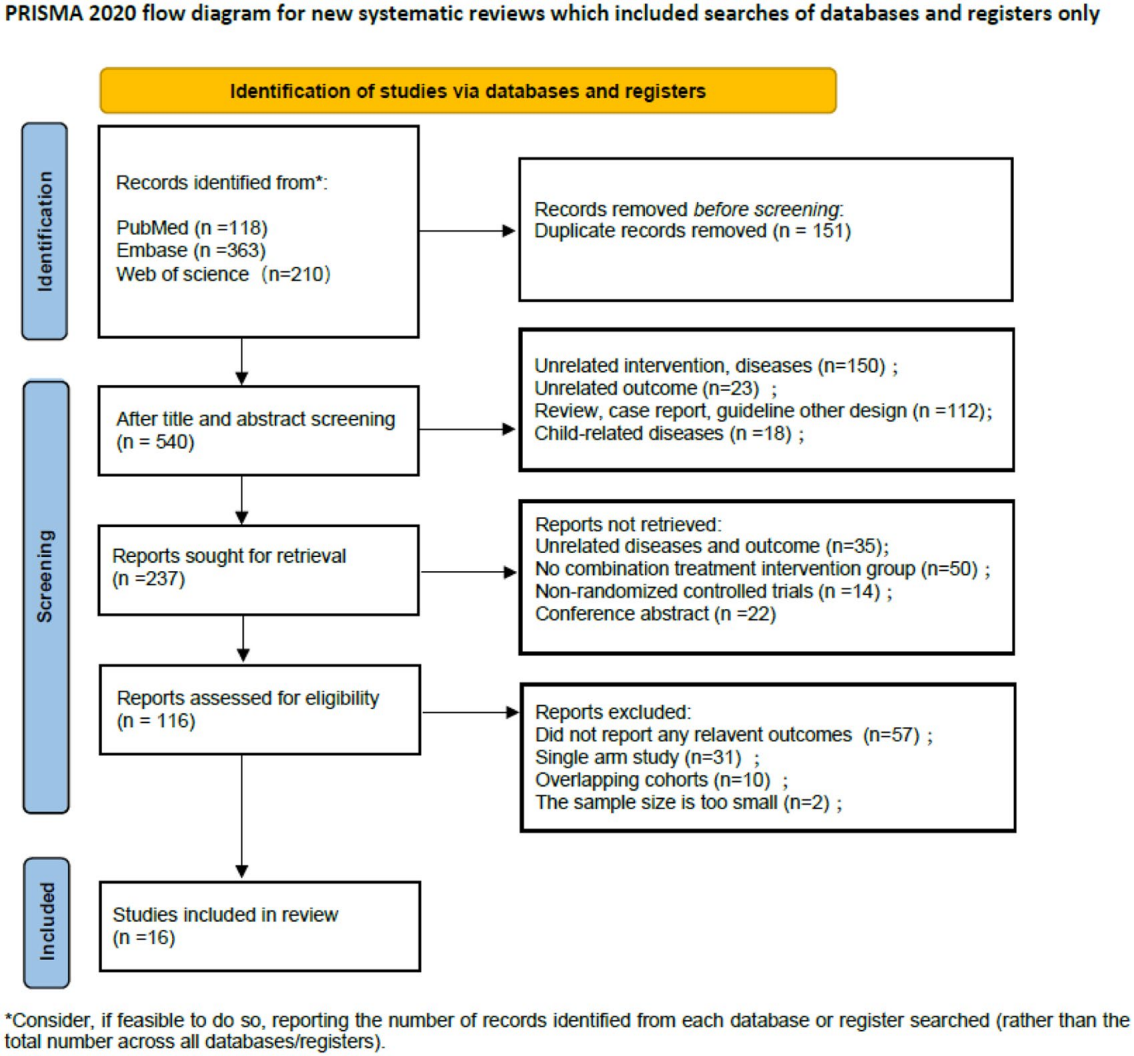
Flow chart of randomized controlled trials for evaluation.

The initial literature search yielded a total of 691 RCTs. Subsequently, the articles were evaluated according to predetermined inclusion and exclusion criteria. The process of research selection, along with the rationale for the exclusion of certain studies, is depicted in Figure 1. Finally, this meta-analysis included 16 studies, one of which may encompass multiple types of treatment strategies. These studies included 1585 patients who received combination therapy, 923 patients who received NAs monotherapy, and 943 patients who received Peg-IFN monotherapy. The functional cure rate achieved by NAs monotherapy was only 0.3% (2/666), the functional cure rate achieved by Peg-IFN monotherapy was 6.1% (40/655), and the functional cure rate in the combination therapy group was 9.9% (116/1169).

### Main characteristics included in the study

Among the 16 RCTs included in this meta-analysis, 13 were multicentre and three were single centre. Among them, five studies included HBeAg positive CHB patients, five studies included HBeAg negative CHB patients, and six studies included both HBeAg positive and negative patients. Two studies showed that Peg-IFN treatment lasted for 26 weeks (<48 weeks) [11,12], and one study did not conduct follow-up [8]. The main characteristics included in the study are presented in Supplementary Tables 1 and 2.

### Risk of bias included in the study

The 16 eligible studies are RCTs. Supplementary Figure 1 shows an example of the risk bias assessment results provided in the Cochrane Systematic Review Handbook. Different colours (green, yellow, red) and symbols (‘+’, ‘?’, ‘-’) can be used in the figure to represent ‘low-risk bias’, ‘unclear’, and ‘high-risk bias’, respectively. Two reviewers independently assessed seven domains: random sequence generation, allocation concealment, blinding of participants/personnel, blinding of outcome assessment, incomplete outcome data, selective reporting, and other biases. The risk of bias in all studies was assessed as low risk. Only one study implemented the blinding of participants and personnel.

## Comparison of the therapeutic effects of NAs combined with Peg-IFN therapy and NAs monotherapy on CHB patients

### Comparison of therapeutic effects between two groups at the end of treatment

**Figure 2.**
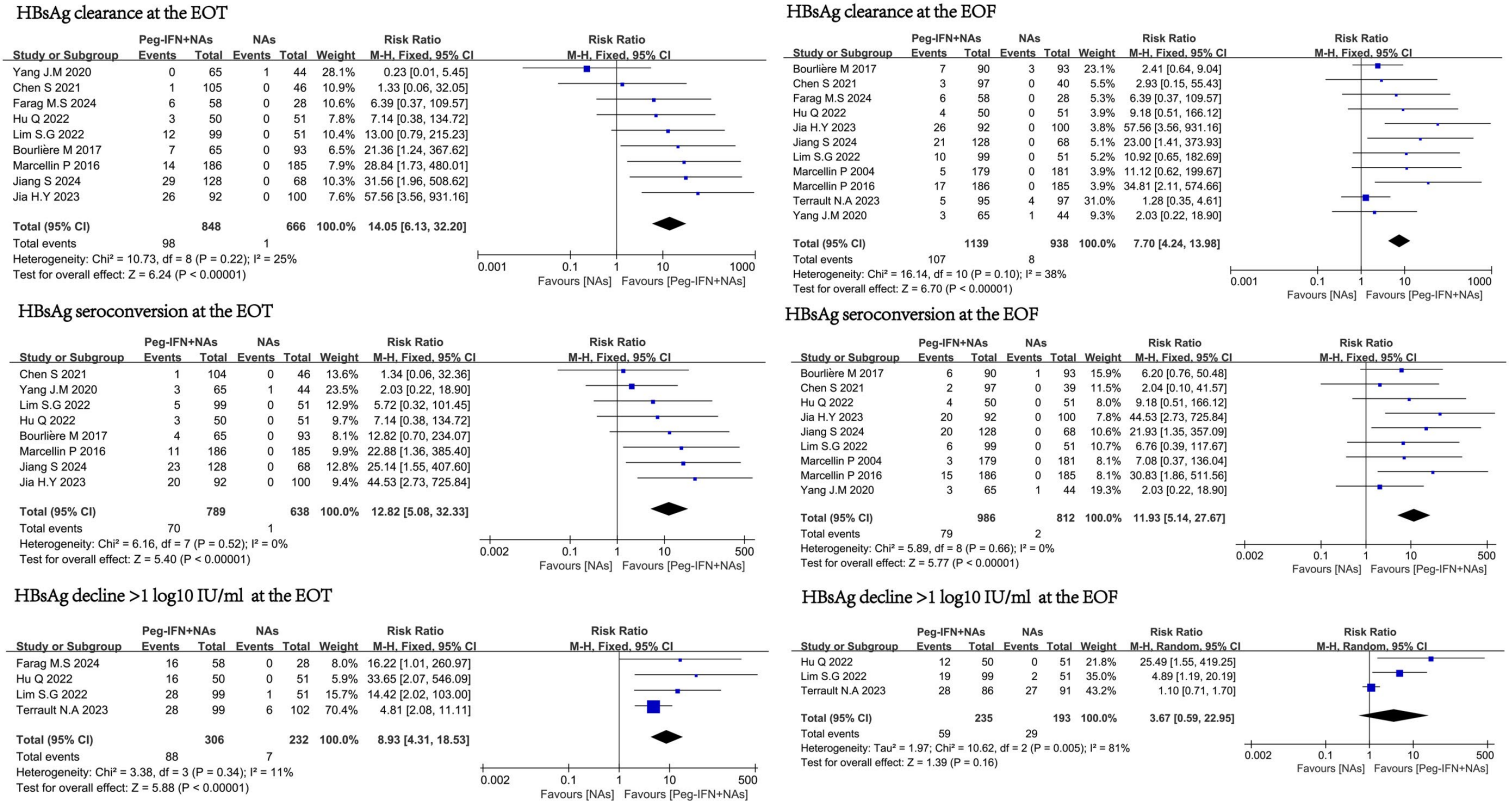
The effects of Peg-IFN combined with NAs therapy and NAs monotherapy on HBsAg clearance rate, HBsAg seroconversion rate, and the rate of qHBsAg reduction > 1 log10 IU/mL.

In this meta-analysis, nine randomized controlled trials compared the efficacy of NAs combined with Peg-IFN treatment group and NAs monotherapy at the EOT [9,11,13–19]. Among them, 9, 8 and 4 studies reported the outcomes of HBsAg clearance rate, HBsAg seroconversion rate, and a rate qHBsAg reduction > 1 log10 IU/mL, respectively. Compared with NAs monotherapy, the combination therapy group achieved a higher HBsAg clearance rate (RR = 14.05, 95% CI 6.13–32.20, I^2^=25%), HBsAg seroconversion rate (RR = 12.82, 95% CI 5.08–32.33, I^2^=0%), and a rate of qHBsAg reduction > 1 log10 IU/mL (RR = 8.93, 95% CI 4.31–18.53, I^2^=11%). (Figure 2)

### Comparison of therapeutic effects between two groups at the end of follow-up

In this meta-analysis, 11 randomized controlled trials compared the efficacy of combination therapy and NAs monotherapy at the EOF [9,11–20]. Among them, 11, 9, and 3 studies reported the outcomes of HBsAg loss rate, HBsAg seroconversion rate, and the rate of qHBsAg reduction > 1 log10 IU/mL, respectively. Compared with NAs monotherapy, the Peg-IFN combined with NAs group achieved a higher HBsAg loss rate (RR = 7.7, 95% CI 4.24–13.98, I^2^=38%), HBsAg seroconversion rate (RR = 11.93, 95% CI 5.14–27.67, I^2^=0%). (Figure 2)

## Comparison of the therapeutic effects of NAs combined with Peg-IFN therapy and Peg-IFN monotherapy on CHB patients

### Comparison of therapeutic effects between two groups at the end of treatment

**Figure 3.**
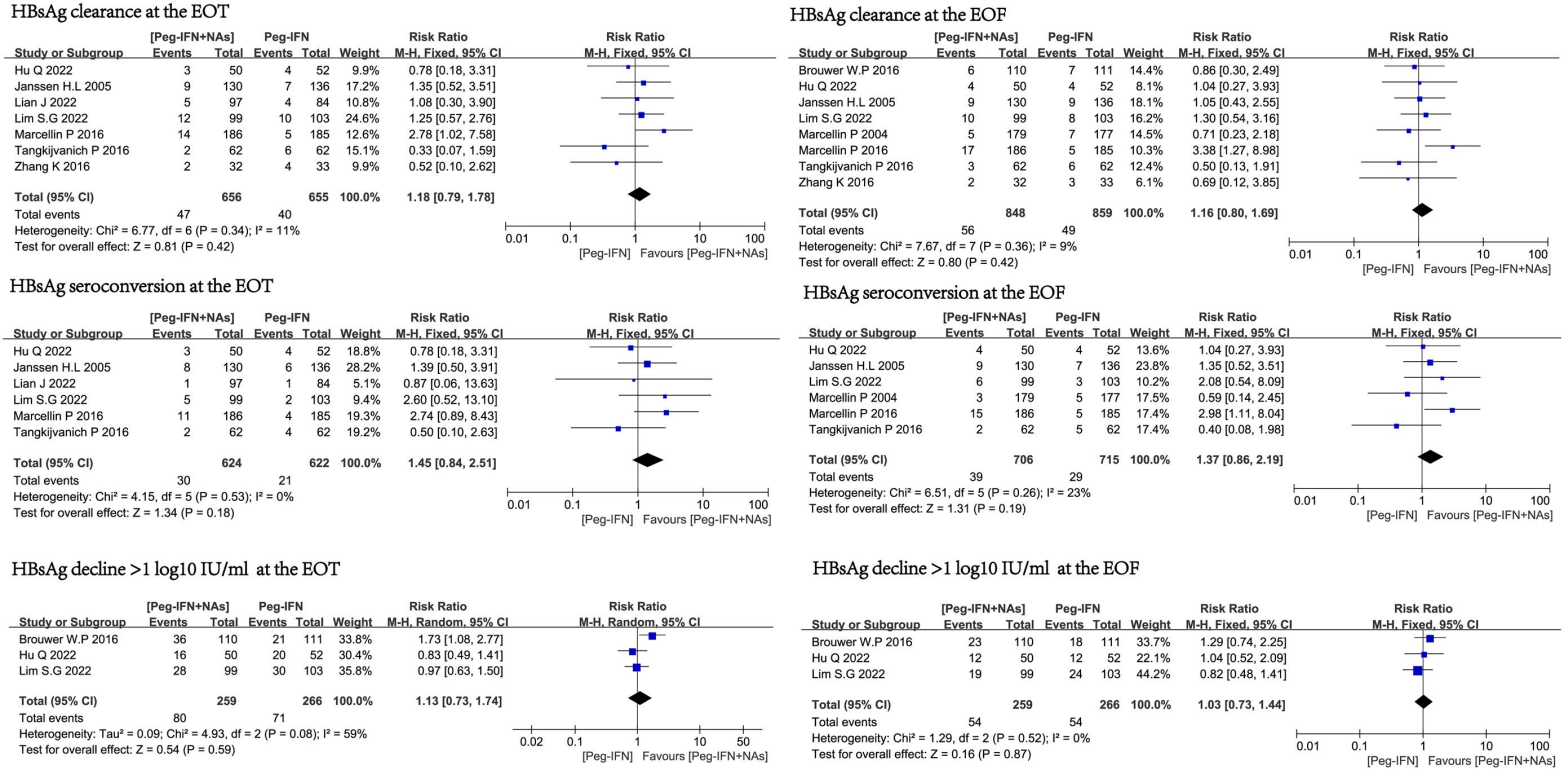
The effects of Peg-IFN combined with NAs therapy and Peg-IFN monotherapy on HBsAg clearance rate, HBsAg seroconversion rate, and the rate of qHBsAg reduction > 1 log10 IU/mL.

In this meta-analysis, seven RCTs compared the efficacy of Peg-IFN combined with NAs group and Peg-IFN monotherapy at the EOT [8,9,14,18,21–23]. Among them, 7, 6, and 3 studies reported the outcomes of HBsAg loss rate, HBsAg seroconversion rate, and the rate of qHBsAg reduction > 1 log10 IU/mL, respectively. Compared with Peg-IFN monotherapy, the combination therapy group did not achieve a higher HBsAg loss rate (RR = 1.18, 95% CI 0.79–1.78, I^2^=11%), HBsAg seroconversion rate (RR = 1.45, 95% CI 0.84–2.51, I^2^=0%), and the rate of qHBsAg reduction > 1 log10 IU/mL (RR = 1.13, 95% CI 0.73–1.74, I^2^=59%) at the EOT (Figure 3).

### Comparison of therapeutic effects between two groups at the EOF

In this meta-analysis, eight RCTs compared the efficacy of Peg-IFN combined with NAs group and Peg-IFN monotherapy at the EOF [9,14,18,20–24]. Among them, 8, 6, and 3 studies reported the outcomes of HBsAg loss rate, HBsAg seroconversion rate, and a rate of qHBsAg reduction > 1 log10 IU/mL, respectively. Compared with Peg-IFN monotherapy, the combination therapy group did not achieve a higher HBsAg loss rate (RR = 1.16, 95% CI 0.80–1.69, I^2^=9%), HBsAg seroconversion rate (RR = 1.37, 95% CI 0.86–2.19, I^2^=23%), and the rate of qHBsAg reduction > 1 log10 IU/mL (RR = 1.03, 95% CI 0.73–1.44, I^2^=0%) at the EOF (Figure 3).

## Subgroup analysis of efficacy comparison between combination therapy group and Peg-IFN monotherapy group

### Subgroup analysis stratified by baseline HBeAg status

To evaluate the impact of baseline HBeAg status (positive or negative) on functional cure in the combination therapy group and Peg-IFN treatment group, we conducted subgroup analysis. At the EOT, regardless of the baseline HBeAg status of CHB patients, the combination therapy group did not achieve higher HBsAg loss rate (combined RR = 1.14, 95% CI 0.69–1.91, I^2^=0%) and HBsAg seroconversion rate (combined RR = 1.12, 95% CI 0.51–2.48, I^2^=0%) compared to the Peg-IFN monotherapy group. At the EOF, regardless of the baseline HBeAg status of CHB patients, the combination therapy group did not achieve higher HBsAg loss rate (combined RR = 1.26, 95% CI 0.86–1.84, I^2^=0%) and HBsAg seroconversion rate (combined RR = 1.33, 95% CI 0.63–2.82, I^2^=0%) compared to the Peg-IFN monotherapy group. (Supplementary Figure 2).

### Subgroup analysis stratified by history of NAs treatment

**Figure 4.**
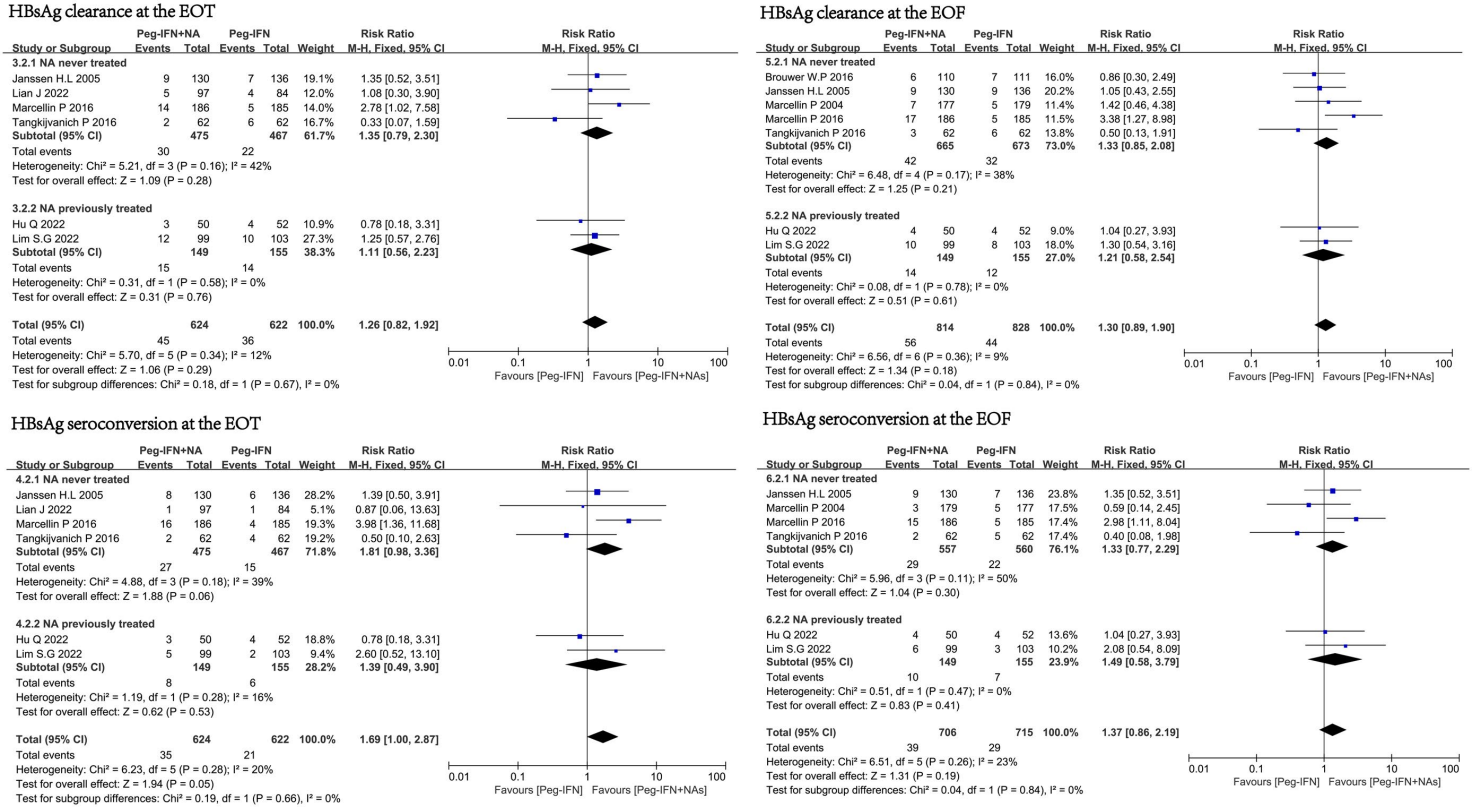
Subgroup analysis stratified by history of NAs treatment. The effects of Peg-IFN combined with NAs therapy and Peg-IFN monotherapy on the outcomes of HBsAg clearance rate and HBsAg seroconversion rate.

At the EOT, regardless of whether CHB patients in the combination therapy group received NAs treatment or initial treatment, compared with the Peg-IFN monotherapy group, the combination therapy group did not achieve higher HBsAg loss rate (combined RR = 1.26, 95% CI 0.82–1.92, I^2^=0%) and HBsAg seroconversion rate (combined RR = 1.69, 95% CI 1.00–2.87, I^2^=20%). At the EOF, compared with the Peg-IFN monotherapy group, the combination therapy group did not achieve higher HBsAg loss rate (combined RR = 1.30, 95% CI 0.89–1.90, I^2^=9%) and HBsAg seroconversion rate (combined RR = 1.37, 95% CI 0.86–2.19, I^2^=23%) (Figure 4).

## Comparison of recurrence risk at the end of follow-up between combination therapy group and Peg-IFN monotherapy group

In this meta-analysis, two RCTs compared the viral and clinical recurrence of Peg-IFN combined with NAs group and Peg-IFN monotherapy at the EOT and EOF [9,14]. Compared with Peg-IFN monotherapy, the combination therapy group had lower virus recurrence rate (RR = 0.14, 95% CI 0.07–0.28, I^2^=30%) and clinical recurrence rate (RR = 0.27, 95% CI 0.08–0.86, I^2^=20%) at the EOT. Similarly, at the EOF, the combination therapy group demonstrated lower virus recurrence rate (RR = 0.08, 95% CI 0.04–0.16, I^2^=0%) and clinical recurrence rate (RR = 0.08, 95% CI 0.01–0.39, I^2^=0%) (Supplementary Figure 3).

## Comparison of safety between combination therapy group and Peg-IFN monotherapy group

**Figure 5.**
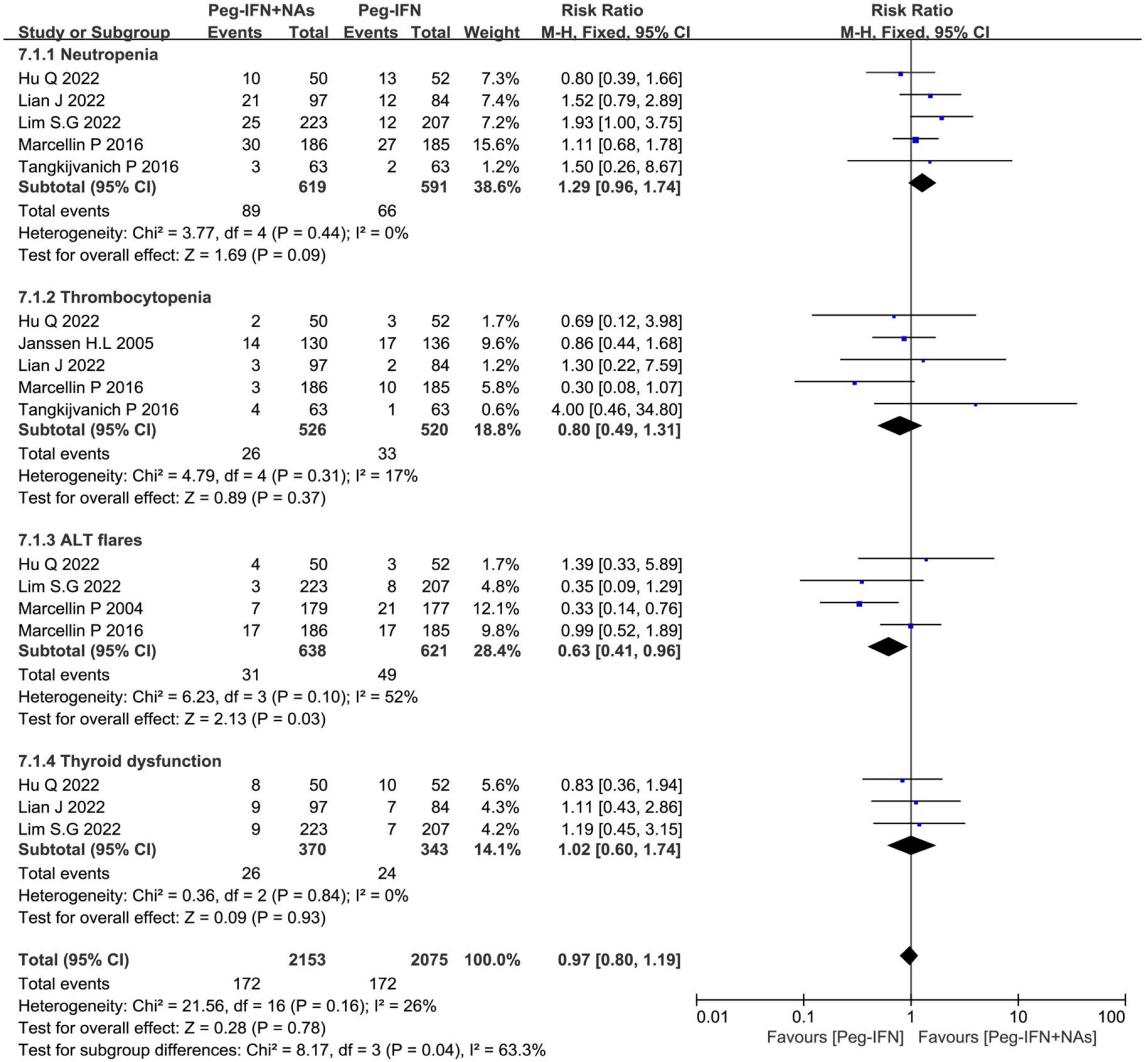
Comparison of safety between combination therapy group and Peg-IFN monotherapy group. Comparison of Peg-IFN combined with NAs therapy and Peg-IFN monotherapy in causing laboratory abnormalities such as neutropenia, thrombocytopenia, ALT flare, and thyroid dysfunction.

Seven trials reported adverse events [8,9,14,18,20–22]. The most common adverse events included influenza-like syndrome, headache, fever, hair loss, rash, gastrointestinal symptoms, Anemia, leukopenia, thrombocytopenia, elevated transaminase, thyroid dysfunction, depression, etc. Most adverse events were mild and resolved with symptomatic treatment. We compared the main laboratory abnormalities observed between the two groups in the incidence of thrombocytopenia (<75 × 10^9^/L), neutropenia (<1.0 × 10^9^/L), or thyroid dysfunction. However, patients in the Peg-IFN monotherapy group had a significantly higher incidence of ALT elevation (>5 × ULN) than those in the combination therapy group (Figure 5).

Furthermore, the incidence of serious adverse events and grade 3 or higher laboratory abnormalities was generally low, with no significant differences observed between the two groups. Detailed data are provided in Supplementary Table 3.

### Sensitivity analysis and publication bias

We conducted a sensitivity analysis comparing the functional cure rates between the combination therapy group and the monotherapy group (NAs or Peg-IFN group) at the EOT or at the EOF. The results showed that after removing the literature one by one, there was no significant change in the merged estimates of the remaining literature (Supplementary Tables 4–11). The meta-analysis results included in the literature are robust and the conclusions are reliable. No publication bias is revealed by visually examining the funnel plot (Supplementary Figures 4 and 5).

## Discussion

Achieving functional cure has become the goal of antiviral therapy for CHB patients. Previous studies have shown that NAs monotherapy or combined therapy with Peg-IFN α can reduce HBV DNA load and achieve HBeAg loss in CHB patients, with a small percentage of patients achieving functional cure [25]. As is known, the mechanism of anti-HBV of NAs is mainly to inhibit the replication of the virus. IFN can directly inhibit viral replication and the transcription of cccDNA through epigenetic modifications [6]. Moreover, IFN can also activate immune responses to exert antiviral effects. Due to the different anti-HBV mechanisms of NAs and IFN, the combination of these two regimens may enable more CHB patients to achieve functional cure. Consequently, we performed a meta-analysis encompassing 16 RCTs to assess the efficacy and safety of various strategies aimed at attaining a functional cure.

Among the studies included in this meta-analysis, a total of 11 studies compared the efficacy of combination therapy and NAs monotherapy, and 9 studies compared the efficacy of combination therapy and Peg-IFN monotherapy. The overall functional cure rate of the combination therapy group was 9.9%, and the overall functional cure rate of Peg-IFN was 6.1%. This meta-analysis found that at the EOT and the EOF, the Peg-IFN and NAs combination therapy group achieved higher HBsAg clearance rate, HBsAg seroconversion rate, and a rate of qHBsAg reduction > 1 log10 IU/mL compared to NAs monotherapy. Two other meta-analyses also confirmed that the combination therapy group can achieve a higher functional cure rate [26,27]. Our findings therefore reinforce the consensus on the benefit of combination therapy over NA monotherapy. However, contrary to the conclusion drawn by Wei et al. [28], our analysis did not prove that the combined therapy has a significant advantage over pegylated interferon monotherapy during the short-term follow-up period. This discrepancy may stem from variations in patient populations, treatment strategies (de novo vs. sequential combination), and follow-up durations. Notably, our exclusive focus on RCTs provides higher-level evidence, and our evaluation of both EOT and EOF responses offers more comprehensive insights into long-term efficacy. The potential advantage of combination therapy is its additional or even synergistic antiviral effect. The reason may be that (1) NAs inhibiting HBV replication may enhance IFN mediated non-specific immunity [29,30]; (2) NAs can rapidly reduce HBV DNA load and alleviate the suppression of HBV specific immune response by high viral load [31]; (3) IFN can promote the innate immune response of CHB patients, especially the antiviral ability of CD56^bright^ NK cells [32].

Furthermore, combination therapy and Peg-IFN monotherapy showed no significant difference in functional cure rates at EOT or EOF. Substantial heterogeneity (I^2^=59%) was observed for qHBsAg decline > 1 log10 IU/mL. Although formal subgroup analysis was precluded by the limited number of studies, descriptive comparisons suggested that heterogeneity arose from differences in treatment history and baseline HBsAg levels. The study by Brouwer et al. involved treatment-naïve patients with higher HBsAg (∼4.4 log10 IU/mL), while others enrolled treatment-experienced patients with lower HBsAg (∼3.5 log10 IU/mL) [24]. Mechanistically, combination therapy may synergize in treatment-naïve patients through NA-induced viral suppression and Peg-IFN-mediated immune activation targeting active cccDNA. In treatment-experienced patients, pre-existing viral suppression, quiescent cccDNA, and immune exhaustion may limit added benefit. Although combination therapy improved early virological response, it did not enhance long-term functional cure, underscoring the impact of baseline characteristics on therapeutic heterogeneity. Previous studies have found that initial combination therapy may have significant advantages in achieving undetectable HBV DNA compared to IFN monotherapy [30], which may be due to the advantage of NAs in the suppression of viral load.

Next, we evaluated the efficacy of combination therapy and Peg-IFN monotherapy stratified by NAs treatment duration and baseline HBeAg status. The results indicate that, regardless of whether patients had been treated with NAs for more than one year prior to Peg-IFN therapy or were treatment-naïve, the combination therapy group did not achieve a higher functional cure rate. Current research has shown that IFN treatment can improve the functional cure rate in HBeAg negative CHB patients. Based on the stratification of HBeAg status (HBeAg positive or negative), and consistent with the overall conclusion, the combination therapy group did not demonstrate a significant advantage in achieving functional cure rate, regardless of HBeAg status. It is worth noting that at the EOT and the EOF, two studies reported virus recurrence rates and clinical recurrence rates between the two groups. Compared with combination therapy, the NAs switching to Peg-IFN monotherapy group showed higher rates of viral recurrence and clinical recurrence. The Peg-IFN monotherapy group showed high rates of viral and clinical recurrence, which may be related to the withdrawal of NAs from treatment. The interpretation of functional cure is limited by varying follow-up durations across studies. While most trials (14/16) had follow-up periods of 72–96 weeks, only one extended to 240 weeks. This variability affects the assessment of sustained response, as shorter follow-up may miss late relapses and potentially overestimate cure rates. Analysis of relapse rates was limited to two studies with 72-week follow-up; the study with 240-week follow-up did not report relapse outcomes. The lack of long-term relapse data precluded meaningful subgroup analysis by follow-up duration. Future studies should standardize extended follow-up periods and comprehensively report relapse events to better evaluate the durability of functional cure.

In this meta-analysis, we also evaluated the safety of combination therapy and Peg-IFN monotherapy in the treatment of CHB. Multiple studies have suggested that Peg-IFN treatment can cause some adverse reactions, including fever, headache, gastrointestinal symptoms, leukopenia, thrombocytopenia, elevated ALT, and thyroid dysfunction. We summarized these laboratory abnormalities and found that the frequency of neutropenia, thrombocytopenia, and thyroid dysfunction caused by the combination therapy group was comparable to that of Peg-IFN monotherapy. However, the Peg-IFN monotherapy group caused more ALT flare compared to the combination therapy group. This comprehensive comparative safety analysis addresses a gap in the existing literature and provides clinicians with valuable insights for treatment decision-making. Previous studies have shown that ALT flare (ALT > 5 × ULN) during Peg-IFNαtreatment is associated with subsequent decreases in HBsAg and HBV RNA, and predicts subsequent HBsAg loss [33]. Moreover, baseline HBsAg levels < 1000 IU/mL, qHBsAg decrease > 0.5 log10 IU/mL at week 12, and sudden increase in ALT (ALT > 3 × ULN) at week 12 were independently associated with good outcomes, i.e. reaching low HBsAg levels and HBsAg disappearance at the end of Peg-IFN treatment at week 48 [34]. Robert et al. found an association between the level of ALT flare and the decrease of HBsAg treatment-related severity, which was more pronounced in HBeAg positive patients [35]. Moreover, compared to patients who did not experience ALT flare, CHB patients with ALT > 5 × ULN during antiviral therapy are more likely to achieve a decrease in qHBsAg > 1 log10 IU/mL [35].

Therefore, the higher incidence of ALT elevation in the monotherapy group should not be interpreted merely as an adverse reaction, but rather as an indicator of active immune-mediated viral clearance, which forms the mechanistic basis for its association with HBsAg decline or disappearance. Studies have shown that Peg-IFN can increase the frequency of HBV-specific T cells and enhance the cytotoxic function of NK cells [36]. These immune cells clear infected hepatocytes and degrade viral cccDNA, a process that results in cellular damage and the release of ALT. Thus, elevated ALT can be regarded as a marker of effective immune targeting of infected cells and is often accompanied by a decrease in HBsAg levels.

In contrast, ALT elevation following discontinuation of NAs is generally not accompanied by a reduction or disappearance of HBsAg and may instead be associated with viral rebound and disease reactivation [37]. This type of ALT elevation, caused by viral resurgence (such as reactivation of cccDNA), represents a ‘passive’ response that lacks effective immune clearance. Consequently, it does not lead to a reduction in HBsAg and may even increase the long-term risk of liver disease—fundamentally differing in both mechanism and clinical significance from Peg-IFN-associated ALT elevation.

In clinical practice, the significance of ALT elevation should be comprehensively evaluated based on whether it is accompanied by effective immune clearance. If ALT elevation occurs alongside a significant decline in HBsAg, it often indicates a beneficial immune response, is associated with a favourable prognosis and can be managed through close monitoring. Conversely, if ALT elevation is not accompanied by a decrease in HBsAg (e.g. after NA discontinuation) or is accompanied by signs of decompensation, it may suggest disease progression or viral rebound, necessitating prompt intervention to prevent severe liver injury.

To verify the reliability of our findings, we evaluated the risk of bias in the 16 included RCTs using the Cochrane Risk of Bias Tool, with an overall judgment of a low risk of bias. This conclusion was supported by a consistent low risk across six key domains (e.g. random sequence generation, allocation concealment, and complete outcome data)—all foundational to internal validity: proper randomization and allocation concealment minimized selection bias, while complete outcome data avoided attrition bias that could distort effect estimates. A key observation concerned the “blinding of participants and personnel”: only 1 study implemented adequate blinding, 1 provided no blinding information, and 14 explicitly did not apply blinding. However, all outcomes in our analysis (including efficacy indicators such as HBsAg clearance and safety indicators such as neutropenia) were laboratory-measured indices, thereby substantially mitigating potential performance/detection bias. These indices relied on standardized instruments rather than subjective judgment; thus, unblinding did not alter measurement accuracy. While blinding remains ideal for RCTs, the objective and standardized nature of our outcome measures ensured the meta-analysis’s reliability despite its absence.

Our meta-analysis also has several limitations. Firstly, factors such as demographic characteristics, baseline HBsAg levels, duration of Peg-IFN therapy, combination therapy approach, and past response of CHB patients to IFN may affect the response to combination therapy. Secondly, the included studies predominantly enrolled Chinese patients infected with HBV genotypes B or C, whereas populations typical of Western regions—who are more frequently infected with genotypes A or D—were underrepresented. HBV genotype and ethnic background may affect treatment outcomes by modulating host immunity and viral behaviour. For example, evidence suggests that in peginterferon-α therapy, patients with genotype B achieve higher early HBeAg seroconversion rates than those with genotype C [38]. Moreover, genotypes A and B are associated with a more substantial decline in HBsAg, while genotype C responds better to add-on therapy than to de novo combination regimens. In contrast, genotype D demonstrates only modest HBsAg reduction regardless of treatment approach. Consequently, the generalizability of our findings to other ethnic or genotypic populations remains uncertain and should be interpreted cautiously [24]. Thirdly, there is limited research on subgroup analysis of some outcomes of interest, and caution should be exercised when interpreting such results. Fourthly, based on limited research, there is a difference in ALT flare between the combination therapy group and Peg-IFN monotherapy group, and it is currently unclear whether this difference is due to an increase in viral load or an enhancement of the body’s immune system. More research is needed to clarify the association between elevated ALT level, viral load, and HBsAg clearance. Finally, based on the limited RCT studies included, it is not yet clear which treatment regimen (NAs initial combined with Peg-IFN treatment versus NAs sequential Peg-IFN treatment) is more effective for functional cure.

Despite these limitations, our study provides important advancements over previous meta-analyses by exclusively focusing on RCTs and evaluating both EOT and EOF responses. Future research should address the identification of optimal candidate populations, refinement of combination strategies and timing, and extension of follow-up duration to confirm the durability of functional cure. The development of predictive models integrating virological, immunological, and clinical features is essential to guide individualized treatment decisions and ultimately improve functional cure rates in CHB.

In summary, the treatment regimen that combines Peg-IFN with NAs demonstrates greater efficacy in enhancing the functional cure rate when compared to NAs monotherapy. However, compared to Peg-IFN monotherapy, the combination therapy group did not benefit more in terms of HBsAg seroconversion rate and HBsAg clearance rate. Compared with combination therapy, Peg-IFN monotherapy showed more ALT flare.

## Supplementary Material

PRISMA_2020_checklist .docx

Supplementary material20250914.docx

## Data Availability

Data are available from the corresponding author upon reasonable request.
