# Supplementary material for: Efficacy of NAs in combination with Peg-IFN for functional cure in patients with CHB: a meta-analysis of RCTs
Source: Ann Med. 2026 Mar 4;58(1):2636319. doi: 10.1080/07853890.2026.2636319 (PMC12961710; doi:10.1080/07853890.2026.2636319)
Supplement: Supplementary material20250914.docx [file IANN_A_2636319_SM9815.docx]

**Efficacy of NAs in Combination with Peg-IFN for Functional Cure in Patients with CHB: A Meta-Analysis of RCTs**

1. **Literature retrieval strategy**

((((("Hepatitis B, Chronic"[Mesh]) OR (((Hepatitis B Virus Infection, Chronic) OR (Chronic Hepatitis B Virus Infection)) OR (Chronic Hepatitis B))) AND (("Interferons"[Mesh]) OR (Interferon))) AND (((Randomized control) OR (Random)) OR (Randomized controlled trial))) AND (((((Hepatitis B Surface Antigens) OR (Australia Antigen)) OR (Antigen, Australia)) OR (HBsAg)) OR (Hepatitis B Surface Antigen))) AND ((((((((lamivudine) OR (Adefovir)) OR (Telbivudine)) OR (Tenofovir)) OR (Tenofovir disoproxil fumarate)) OR (Tenofovir alafenamide fumarate)) OR (Tenofovir amibufenamide)) OR (Entecavir))

1. **Risk of bias included in the study**

**
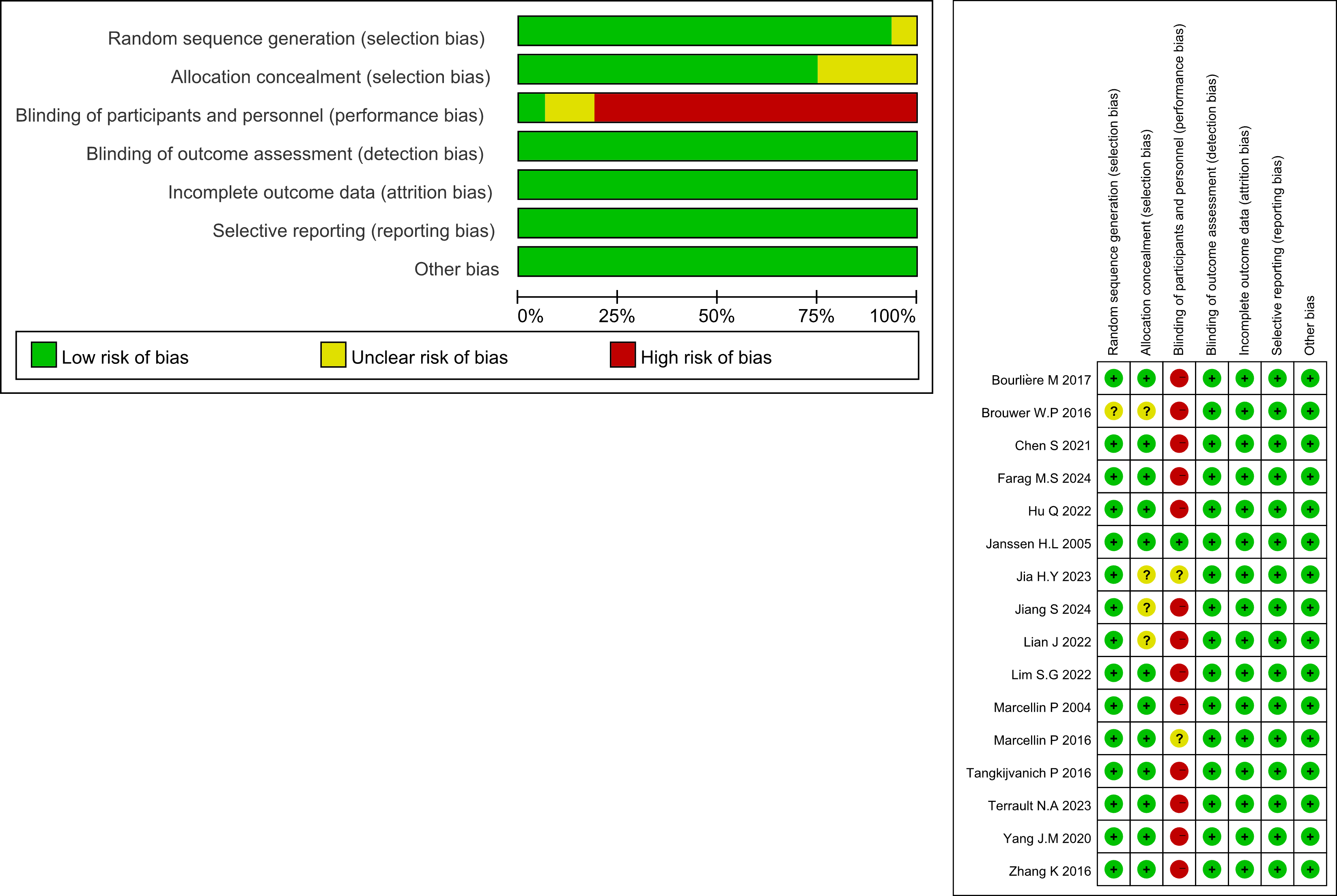
**

**Supplementary Figure 1**. **Quality assessment of randomized control trials**

**Subgroup analysis of efficacy comparison between combination therapy group and Peg-IFN monotherapy group**

**Subgroup analysis stratified by baseline HBeAg status**

**
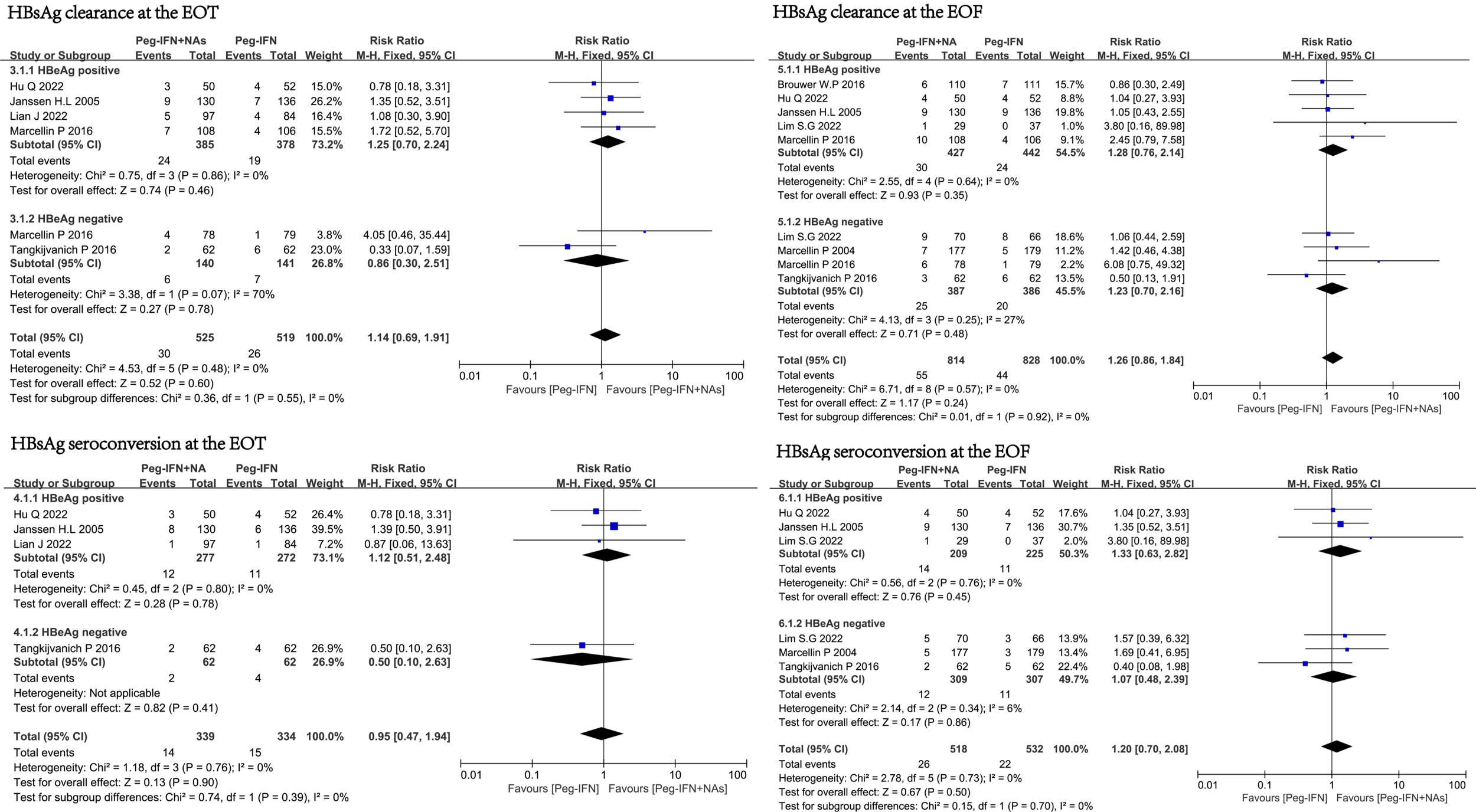
**

**Supplementary Figure 2. Subgroup analysis stratified by HBeAg status. The effects of Peg-IFN combined with NAs therapy and Peg-IFN monotherapy on HBsAg clearance rate and HBsAg seroconversion rate outcomes**

**Comparison of recurrence risk at the end of follow-up between combination therapy group and Peg-IFN monotherapy group**

**
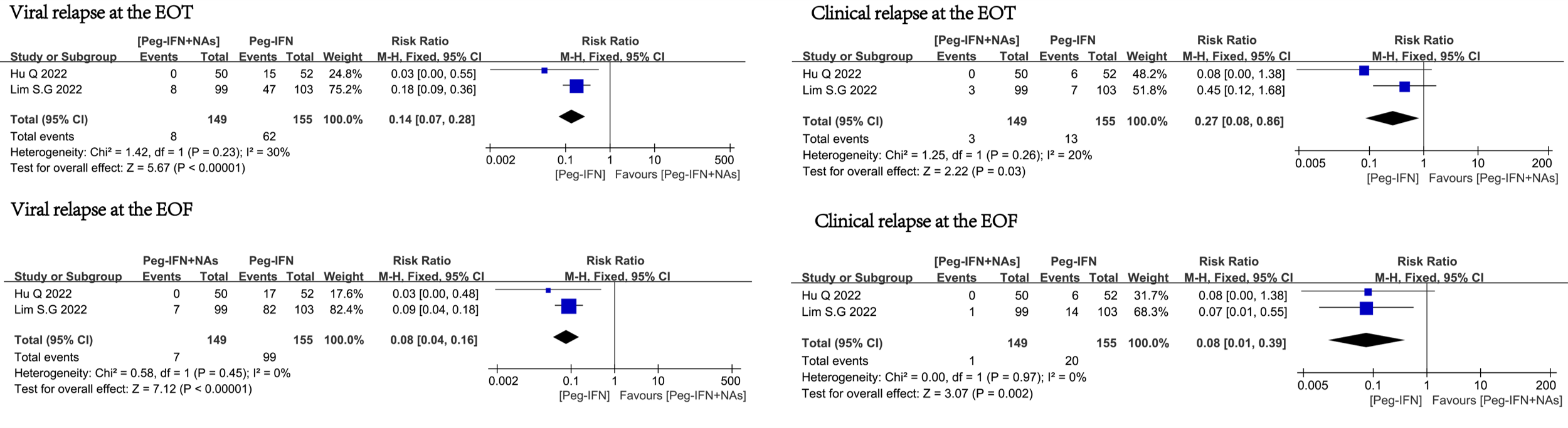
**

**Supplementary Figure 3. Comparison of virus recurrence and clinical recurrence risk between Peg-IFN combined with NAs therapy and Peg-IFN monotherapy in CHB patients at the EOT and the EOF.**

1. **Funnel plot**


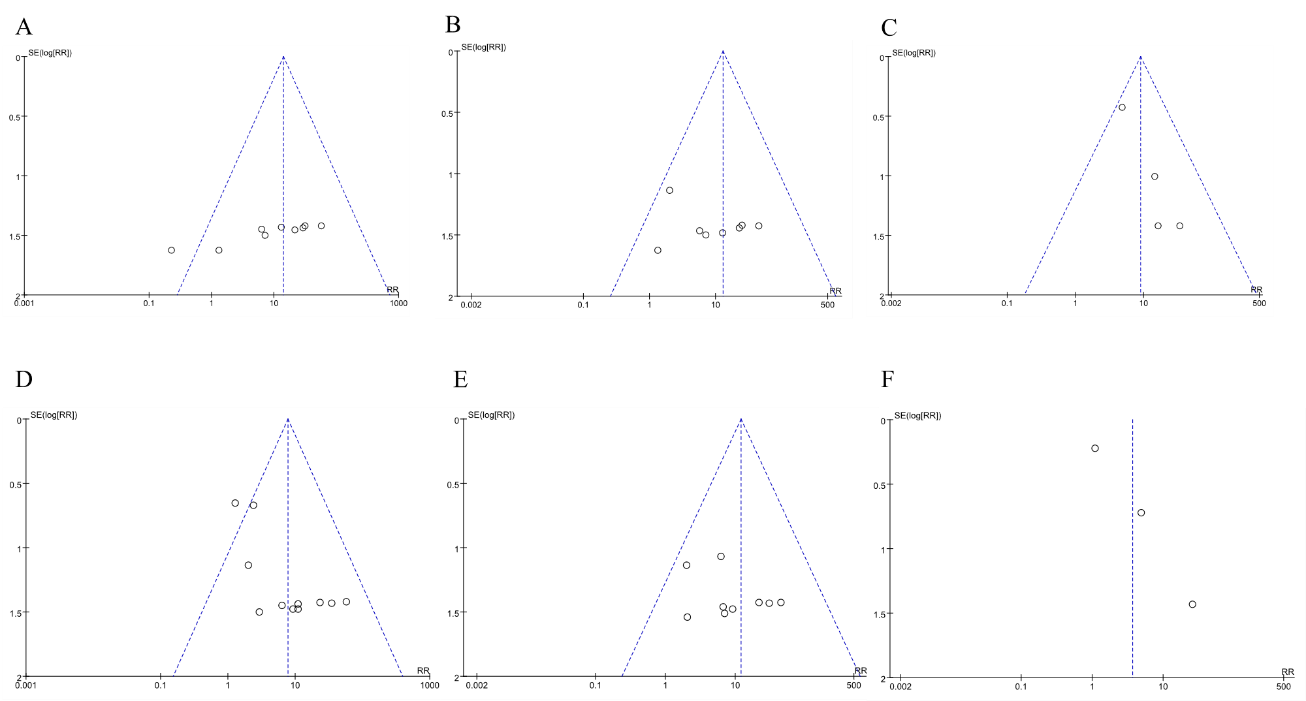


**Supplementary Figure 4: Funnel plot of effectiveness between the combination therapy group and the NAs monotherapy group at the EOT and the EOF.** At the EOT: A. HBsAg Clearance rate, B.HBsAg seroconversion rate, C. a rate of qHBsAg decrease >1 log10 IU/mL. At the EOF: D. HBsAg Clearance rate, E. HBsAg seroconversion rate, F. a rate of qHBsAg decrease>1 log10 IU/mL.


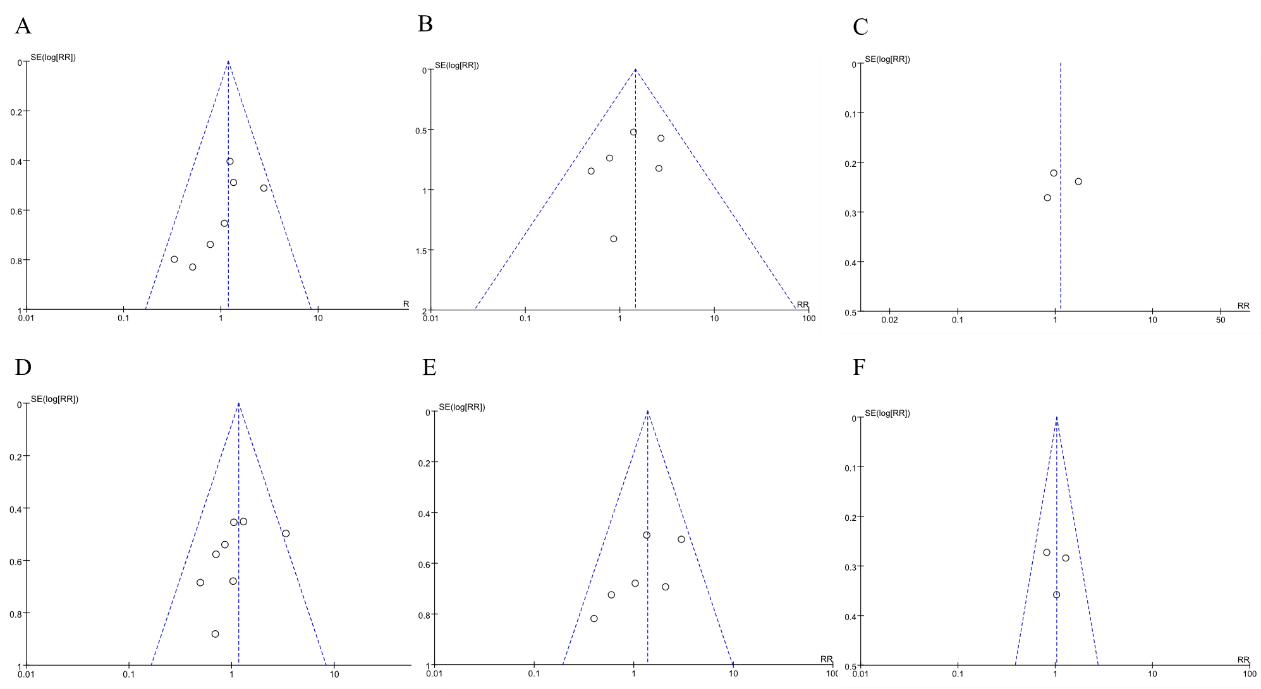


**Supplementary Figure 5: Funnel plot of the effectiveness of the combination therapy group and Peg-IFN monotherapy group at the EOT and the EOF.** At the EOT: A. HBsAg Clearance rate, B.HBsAg seroconversion rate, C. a rate of qHBsAg decrease >1 log10 IU/mL. At the EOF: D. HBsAg Clearance rate, E. HBsAg seroconversion rate, F. a rate of qHBsAg decrease>1 log10 IU/mL.

1. **Main characteristics included in the study**

| **Supplementary Table 1. Characteristics of all included RCT studies** | | | | | | | | | | |
| --- | --- | --- | --- | --- | --- | --- | --- | --- | --- | --- |
| Study | Region | Design | Total sample size（n） | IFN+NAs  （n） | IFN  （n） | NAs  （n） | HBeAg positive（n） | Previous NA treatment  Time（Weeks） | Peg-IFN treatment period (Weeks) (Dose) | Follow-up period (Weeks) |
| Jiang S 2024 | China | Multicenter RCT | 196 | 128 | N/A | 68 | 81 | ≥24 | 48 (Peg-IFNα-2b, 180 μg/weekly) | 96 |
| Farag M.S 2024 | Netherlands  and Canada | Multicenter RCT | 86 | 58 | N/A | 28 | 0 | ≥48 | 48 (Peg-IFNα-2a,  180 μg/weekly) | 72 |
| Terrault N.A 2023 | United States and Canada | Multicenter RCT | 201 | 99 | N/A | 102 | 103 | 0 | 24 (Peg-IFNα-2a,  180 μg/weekly) | 240 |
| Jia H.Y 2023 | China | Multicenter RCT | 192 | 92 | N/A | 100 | 0 | ＞48 | 48 (Peg-IFNα-2b  180 μg/weekly) | 72 |
| Lim S.G 2022 | Singapore | Multicenter RCT | 253 | 99 | 103 | 51 | 82 | ＞48 | 48 (Peg-IFNα-2b 1.5ug/kg/weekly) | 72 |
| Lian J 2022 | China | Multicenter RCT | 181 | 97 | 84 | N/A | 181 | 0 | 48 (Peg-IFNα-2b, 180 μg/weekly) | N/A |
| Hu Q 2022 | China | Multicenter RCT | 153 | 50 | 52 | 51 | 153 | ＞96 | 48 (Peg-IFNα-2b, 180 μg/weekly) | 72 |
| Chen S 2021 | China | Multicenter RCT | 155 | 108 | N/A | 47 | 113 | 26 | 52 (Peg-IFNα-2a,  180 μg/weekly) | 78 |
| Yang J.M 2020 | China | Single center RCT | 109 | 65 | N/A | 44 | 60 | 0 | 26 (Peg-IFNα-2a,  weekly) | 78 |
| Bourlière M 2017 | France | Multicenter RCT | 183 | 90 | N/A | 93 | 0 | ＞48 | 48 (Peg-IFNα-2a,  180 μg/weekly) | 96 |
| Tangkijvanich P 2016 | Thailand | Single center RCT | 124 | 62 | 62 | N/A | 0 | 0 | 48 (Peg-IFNα-2b 1.5ug/kg/weekly) | 96 |
| Brouwer W.P 2016 | 15 countries | Multicenter RCT | 221 | 110 | 111 | N/A | 221 | 0 | 52 (Peg-IFNα-2b,  100 μg/weekly) | 78 |
| Marcellin P 2016 | 19 countries | Multicenter RCT | 556 | 186 | 185 | 185 | 323 | 0 | 48 (Peg-IFNα-2a  180 μg/weekly) | 72 |
| Zhang K 2016 | China | Single center RCT | 65 | 32 | 33 | N/A | 65 | 0 | 48(peg-IFN alfa-2a  135 μg weekly) | 96 |
| Marcellin P 2004 | 13 countries | Multicenter RCT | 537 | 179 | 177 | 181 | 0 | 0 | 48 (Peg-IFNα-2a,  180 μg/weekly) | 72 |
| Janssen H.L 2005 | 15 countries | Multicenter RCT | 266 | 130 | 136 | N/A | 266 | 0 | 52 (Peg-IFNα-2b,  100 μg/weekly) | 78 |

The follow-up period is defined as the entire duration from treatment initiation to the end of the study, encompassing both on-treatment and off-treatment observation

| **Supplementary Table 2. Baseline patient characteristics** | | | | | | |
| --- | --- | --- | --- | --- | --- | --- |
|  | HBsAg level，log_10_ IU/mL | | | ALT level（U/L） | | |
| Study | IFN+NAs | IFN | NAs | IFN+NAs | IFN | NAs |
| Jiang S 2024 | 3.07 (2.35, 3.46) |  | 3.04 (2.65, 3.36) | 26.0 (19.0, 34.0) |  | 25.5 (19.0, 31.5) |
| Farag M.S 2024 | 2.6 (1.0) |  | 2.6 (0.9) | 28 (16) |  | 28 (12) |
| #Terrault N.A 2023 | 3.6 (3.0, 4.9) |  | 3.9 (3.0, 4.5) | 75 (57,117)  66.5 (47.5, 117.5) |  | 82 (59, 135)  71 (49, 129) |
| Jia H.Y 2023 | 2.74 (0.47) |  | 2.71（0.69） | 24.00 (8.75) |  | 23.75（9.43） |
| Lim S.G 2022 | 2.85 (2.26–3.33) | 3.03(2.65–3.33) | 2.86(2.65–3.35) | 66 (77–95) | 68 (78–93) | 66 (81–100) |
| Lian J 2022 | 4.12 (0.54) | 3.96 (0.67) |  | 132 (89−199) | 118 (88−192) |  |
| Hu Q 2022 | 3.24 (2.95–3.39) | 3.19 (2.92–3.39) | 3.17 (2.97–3.32) | 21 (16–38) | 24 (15–30) | 17 (12–29) |
| Chen S 2021 | 3.7 (3.3, 4.2) |  | 3.7 (3.3, 4.2) | 83.1 (46.0, 161.9) |  | 79.2 (49.5, 164.0) |
| Yang J.M 2020 | 3.30（2.92,3.95） |  | 3.26 (2.82,3.71) | 50.0 (30.8,107.5) |  | 59.0 (43.0,100.5) |
| Bourlière M 2017 | 3.0（0.7） |  | 2.9 (0.9) | 35 (21) |  | 33 (15) |
| Tangkijvanich P 2016 | 3.5 (0.5) | 3.4 (0.5) |  | 72.5 (40.0) | 75.5 (32.2) |  |
| Brouwer W.P 2016 | 4.4 (0.7) | 4.4 (0.6) |  | 168 (120) | 172 (120) |  |
| Marcellin P 2016 | 3.9 (0.8) | 3.8 (0.8) | 3.9 (0.8) | 121 (181) | 107 (92) | 101 (68) |
| Zhang K 2016 |  |  |  | 156 (96) | 172 (104) |  |
| Marcellin P 2004 |  |  |  | 90.8 (76.2) | 94.4 (85.9) | 105.7 (128.2) |
| Janssen H.L 2005 |  |  |  | 176 (156) | 173 (124) |  |

ALT, alanine aminotransferase; ULN, upper limit of normal; The ULN for ALT was defined as 40 U/L.

ALT, # Male and female

| **Supplementary Table 3. Serious Adverse Events and laboratory abnormalities** | | | | | | |
| --- | --- | --- | --- | --- | --- | --- |
| SAEs Category | Combination Therapy (n/N, %) | Peg-IFN Monotherapy (n/N, %) | | P Value | Study Reference | |
| *Serious adverse events | 2/50 (4%) | 3/52 (5.8%） | | 1.000 | HU Q 2022 | |
| *Serious adverse events | 21/186(11%） | 18/185 (10%） | | 0.746 | Marcellin P 2016 | |
| *Serious adverse events | 12/179(7%） | 9/177 (5%) | | 0.662 | Marcellin P 2004 | |
| Serious thyroid disorders | 0 | 2/177 (1%) | | 0.497 | Marcellin P 2004 | |
| Death | 0 | 1/177 (0.6%) | | 1.000 | Marcellin P 2004 | |
| Neutropenia  (＜1.0×10⁹/L) | 21/97 (21.65%） | 12/84 (14.29%) | | 0.238 | Lian J 2022 | |
| Neutropenia  （＜1.0×10⁹/L） | 10/50 (20%） | 13/52 (25%) | | 0.643 | Hu Q 2022 | |
| Neutropenia  （＜1.0×10⁹/L） | 30/186 (16%) | 27/185 (15%) | | 0.781 | Marcellin P 2016 | |
| Neutropenia  （＜0.75×10⁹/L） | 3/63 (4.8%） | 2/63 (3.2%) | | 1.000 | Tangkijvanich P 2016 | |
| Neutropenia  （＜0.75×10⁹/L） | 22/223 (9.9%) | 12/207 (5.8%) | | 0.118 | Lim S.G 2022 | |
| Neutropenia  （＜0.5×10⁹/L） | 3/223 (1.3%) | 0 | | 0.248 | Lim S.G 2022 | |
| Neutropenia  （＜0.5×10⁹/L） | 2/97 (2%) | 1/84 (1.2%) | 1.000 | | | Lian J 2022 |
| Thrombocytopenia  （＜50×10⁹/L） | 3/97 (3.09%） | 2/84 (2.38%) | 1.000 | | | Lian J 2022 |
| Thrombocytopenia  （＜50×10⁹/L） | 2/50 (4%) | 3/52 (5.8%) | 1.000 | | | Hu Q 2022 |
| Thrombocytopenia  （＜50×10⁹/L） | 3/186 (1.6%) | 10/185 (5.4%) | 0.048 | | | Marcellin P 2016 |
| ALT＞5 × ULN | 4/50 (8%) | 3/52 (5.8%) | 0.714 | | | Hu Q 2022 |
| ALT＞5 × ULN | 3/223 (1.3%) | 8/207 (3.9%) | 0.137 | | | Lim S.G 2022 |
| ALT＞10× ULN | 0 | 1/207 (0.5%) | 0.484 | | | Lim S.G 2022 |
| ALT＞10× ULN | 7/179 (4%) | 21/177 (12%) | 0.004 | | | Marcellin P 2004 |
| ALT＞10× ULN | 17/186 (9%) | 17/185 (9%) | 1.000 | | | Marcellin P 2016 |

* A serious adverse event was one that presented a clinically significant hazard or resulted in contraindication or side effect.

1. **Sensitivity analysis**

**Supplementary** **Table 4.** **Sensitivity analysis of HBsAg clearance rate comparison between the combination therapy group and the NAs monotherapy group at the EOT**

| Culling study | RR | 95% CI | I^2^ | P |
| --- | --- | --- | --- | --- |
| Jiang S 2024 | 12.04 | 5.12，28.33 | 34% | ＜0.00001 |
| Farag M.S 2024 | 14.95 | 6.25，35.76 | 36% | ＜0.00001 |
| Lim S.G 2022 | 14.17 | 5.95，33.73 | 35% | ＜0.00001 |
| Hu Q 2022 | 14.63 | 6.16，34.73 | 34% | ＜0.00001 |
| Chen S 2021 | 15.61 | 6.50，37.48 | 20% | ＜0.00001 |
| Yang J.M 2020 | 19.45 | 7.36，51.39 | 0% | ＜0.00001 |
| Bourlière M 2017 | 13.54 | 5.68，32.28 | 33% | ＜0.00001 |
| Marcellin P 2016 | 12.78 | 5.34，30.56 | 31% | ＜0.00001 |
| Jia H.Y 2023 | 10.49 | 4.30，25.58 | 20% | ＜0.00001 |

**Supplementary** **Table 5.** **Sensitivity analysis of HBsAg seroconversion rate comparison between combination therapy group and NAs monotherapy group at the EOT**

| Culling study | RR | 95% CI | I^2^ | P |
| --- | --- | --- | --- | --- |
| Jiang S 2024 | 11.00 | 4.20，28.84 | 0% | ＜0.00001 |
| Lim S.G 2022 | 13.87 | 5.19，37.11 | 1% | ＜0.00001 |
| Hu Q 2022 | 13.43 | 5.07，35.58 | 2% | ＜0.00001 |
| Chen S 2021 | 14.62 | 5.43，39.39 | 0% | ＜0.00001 |
| Yang J.M 2020 | 16.13 | 5.64，46.08 | 0% | ＜0.00001 |
| Bourlière M 2017 | 12.82 | 4.84，33.93 | 3% | ＜0.00001 |
| Marcellin P 2016 | 11.72 | 4.38，31.31 | 0% | ＜0.00001 |
| Jia H.Y 2023 | 9.51 | 3.48，25.99 | 0% | ＜0.0001 |

**Supplementary** **Table 6. Sensitivity analysis of HBsAg clearance rate comparison between the combination therapy group and the NAs monotherapy group at the EOF**

| Culling study | RR | 95% CI | I^2^ | P |
| --- | --- | --- | --- | --- |
| Jiang S 2024 | 6.87 | 3.75，12.60 | 37% | ＜0.00001 |
| Farag M.S 2024 | 7.77 | 4.22，14.31 | 45% | ＜0.00001 |
| Lim S.G 2022 | 7.52 | 4.09，13.83 | 43% | ＜0.00001 |
| Hu Q 2022 | 7.64 | 4.15，14.06 | 44% | ＜0.00001 |
| Chen S 2021 | 7.98 | 4.33，14.71 | 44% | ＜0.00001 |
| Yang J.M 2020 | 8.28 | 4.44，15.46 | 42% | ＜0.00001 |
| Bourlière M 2017 | 9.29 | 4.72，18.28 | 38% | ＜0.00001 |
| Marcellin P 2004 | 7.56 | 4.11，13.92 | 43% | ＜0.00001 |
| Marcellin P 2016 | 6.59 | 3.55，12.22 | 32% | ＜0.00001 |
| Jia H.Y 2023 | 5.75 | 3.06，10.79 | 18% | ＜0.00001 |
| Terrault N.A 2023 | 10.59 | 5.21，21.53 | 12% | ＜0.00001 |

**Supplementary** **Table 7. Sensitivity analysis of HBsAg seroconversion rate comparison between the combination therapy group and the NAs monotherapy group at the EOF**

| Culling study | RR | 95% CI | I^2^ | P |
| --- | --- | --- | --- | --- |
| Jiang S 2024 | 10.74 | 4.49，25.71 | 0% | ＜0.00001 |
| Lim S.G 2022 | 12.54 | 5.18，30.36 | 0% | ＜0.00001 |
| Hu Q 2022 | 12.17 | 5.05，29.30 | 0% | ＜0.00001 |
| Chen S 2021 | 13.21 | 5.42，32.19 | 0% | ＜0.00001 |
| Yang J.M 2020 | 14.30 | 5.63，36.29 | 0% | ＜0.00001 |
| Bourlière M 2017 | 13.01 | 5.19，32.60 | 0% | ＜0.00001 |
| Marcellin P 2004 | 12.35 | 5.13，29.71 | 0% | ＜0.00001 |
| Marcellin P 2016 | 10.25 | 4.21，24.95 | 0% | ＜0.00001 |
| Jia H.Y 2023 | 9.18 | 3.72，22.62 | 0% | ＜0.00001 |

**Supplementary** **Table 8. Sensitivity analysis of HBsAg clearance rate comparison between the combination therapy group and Peg-IFN monotherapy group at the EOT**

| Culling study | RR | 95% CI | I^2^ | P |
| --- | --- | --- | --- | --- |
| Lim S.G 2022 | 1.16 | 0.72，1.87 | 26% | 0.53 |
| Lian J 2022 | 1.20 | 0.78，1.84 | 26% | 0.41 |
| Hu Q 2022 | 1.23 | 0.80，1.88 | 22% | 0.34 |
| Tangkijvanich P 2016 | 1.34 | 0.87，2.05 | 25% | 0.54 |
| Janssen H.L 2005 | 1.15 | 0.73，1.81 | 0% | 0.75 |
| Marcellin P 2016 | 0.95 | 0.60，1.51 | 0% | 0.84 |
| Zhang K 2016 | 1.26 | 0.82，1.92 | 12% | 0.29 |

**Supplementary Table 9.** **Sensitivity analysis of HBsAg seroconversion rate comparison between the combination therapy group and Peg-IFN monotherapy group at the EOT**

| Culling study | RR | 95% CI | I^2^ | P |
| --- | --- | --- | --- | --- |
| Lim S.G 2022 | 1.33 | 0.74，2.39 | 0% | 0.34 |
| Lian J 2022 | 1.48 | 0.85，2.59 | 0% | 0.17 |
| Hu Q 2022 | 1.61 | 0.89，2.91 | 0% | 0.12 |
| Tangkijvanich P 2016 | 1.68 | 0.93，3.03 | 0% | 0.09 |
| Janssen H.L 2005 | 1.47 | 0.77，2.80 | 4% | 0.24 |
| Marcellin P 2016 | 1.15 | 0.61，2.17 | 0% | 0.68 |

**Supplementary Table 10. Sensitivity analysis of HBsAg clearance rate comparison between the combination therapy group and Peg-IFN monotherapy group at the EOF**

| Culling study | RR | 95% CI | I^2^ | P |
| --- | --- | --- | --- | --- |
| Lim S.G 2022 | 1.14 | 0.76, 1.71 | 21% | 0.54 |
| Hu Q 2022 | 1.17 | 0.80, 1.73 | 22% | 0.42 |
| Tangkijvanich P 2016 | 1.26 | 0.85, 1.86 | 2% | 0.25 |
| Janssen H.L 2005 | 1.19 | 0.79, 1.79 | 21% | 0.41 |
| Brouwer W.P 2016 | 1.21 | 0.82, 1.81 | 19% | 0.34 |
| Marcellin P 2004 | 1.24 | 0.84, 1.84 | 14% | 0.28 |
| Marcellin P 2016 | 0.91 | 0.60，1.38 | 0% | 0.65 |
| Zhang K 2016 | 1.19 | 0.82，1.75 | 18% | 0.36 |

**Supplementary Table 11. Sensitivity analysis of HBsAg seroconversion rate comparison between the combination therapy group and Peg-IFN monotherapy group at the EOF**

| Culling study | RR | 95% CI | I^2^ | P |
| --- | --- | --- | --- | --- |
| Lim S.G 2022 | 0.88 | 0.48, 1.62 | 34% | 0.32 |
| Hu Q 2022 | 1.42 | 0.86 ,2.35 | 37% | 0.17 |
| Tangkijvanich P 2016 | 1.57 | 0.95, 2.60 | 1% | 0.08 |
| Janssen H.L 2005 | 1.38 | 0.80, 2.36 | 39% | 0.24 |
| Marcellin P 2004 | 1.53 | 0.93, 2.54 | 20% | 0.10 |
| Marcellin P 2016 | 1.03 | 0.59，1.78 | 0% | 0.92 |
